# Eucalyptus Plantation Management Shapes Roe Deer Site-Use Patterns

**DOI:** 10.3390/ani16111613

**Published:** 2026-05-26

**Authors:** Guilherme Ares-Pereira, Rita Tinoco Torres, Daniela Teixeira, Rui G. Morgado, Jorge F. Henriques, Guilherme Castro, Ana Magalhães, Cátia Lima, Cláudia Camarinha, Luís Miguel Rosalino

**Affiliations:** 1 Centre for Ecology, Evolution and Environmental Changes (CE3C), CHANGE—Global Change and Sustainability Institute, Faculdade de Ciências, Universidade de Lisboa, 1749-016 Lisboa, Portugallmrosalino@ciencias.ulisboa.pt (L.M.R.); 2 Centre for Environmental and Marine Studies (CESAM), Department of Biology, University of Aveiro, Campus Universitário de Santiago, 3810-193 Aveiro, Portugal; rita.torres@ua.pt (R.T.T.); danielateixeira9@ua.pt (D.T.); ruimorgado@ua.pt (R.G.M.); jfhenriques@ua.pt (J.F.H.);

**Keywords:** forestry, *Capreolus capreolus*, disturbance, conservation, camera trap, occupancy patterns

## Abstract

The expansion of Eucalyptus plantations has raised concerns about their effects on wildlife, yet the influence of stand-level management on roe deer remains poorly understood. We investigated how plantation management shapes roe deer responses in central Portugal using 375 camera-trap deployments from 2019 and 2020 and four session-specific single-season occupancy models that separated detection probability from site-use probability. Across sessions, stand size was retained mainly in the detection component, indicating that variation in camera-trap sampling coverage influenced detectability more consistently than ecological site use. Support for site-use effects varied among periods, but the strongest result emerged in the 2020 dry season, when site use was lower in reforestation stands than in afforestation stands and temporal responses differed among production regimens. The 2020 wet season also supported a positive effect of time since intervention on site use after accounting for broad spatial structure. By contrast, the 2019 wet season informed detection only, whereas the 2019 dry season showed non-linear relationships weakened by overdispersion and QAICc sensitivity. Overall, our findings indicate that Eucalyptus plantations should not be treated as ecologically uniform systems, because roe deer responses depend on when and how stands are managed, with reforestation representing the most disruptive phase.

## Simple Summary

Eucalyptus plantations cover large areas of central Portugal, but their effects on wildlife depend on how they are managed. We studied how roe deer responded to different stages of plantation management by analyzing records from camera traps placed in five plantation landscapes between 2019 and 2020. We found that roe deer use of plantation areas differed among management stages. The clearest pattern was that reforestation, which involves stronger disturbance such as stump removal, soil preparation, and heavy machinery, was linked to lower roe deer use of plantation areas. We also found evidence that plantation areas may become more suitable again as time passes after intervention, suggesting that roe deer can return as vegetation recovers. In contrast, plantation size mainly influenced how easily roe deer were detected by cameras, rather than whether the animals were truly using the area. These results show that Eucalyptus plantations are not all the same for wildlife and suggest that limiting large, intensive reforestation operations, while maintaining less disturbed areas within plantation landscapes, may help make production forests more compatible with roe deer conservation.

## 1. Introduction

Forestry plantations have become increasingly important in addressing the growing demand for forest products while helping to reduce pressure on natural forests, but they can also alter habitat structure, landscape composition, and biodiversity, particularly when dominated by fast-growing exotic trees [1,2,3]. Among these systems, Eucalyptus plantations have expanded widely outside their native range because of their high economic value and rapid growth rates and are now among the most widespread planted forest systems worldwide [4,5,6]. As a result, these plantations have become an important focus of ecological research aimed at understanding their effects on local ecosystems and biodiversity [7,8].

In Central Portugal (SW Europe), Eucalyptus plantations are widespread and form a major component of production-forest landscapes, providing substantial economic benefits but also posing biodiversity conservation challenges by altering wildlife spatial and temporal patterns [9]. These plantations are characterized by spatial homogeneity, because single-species stands are often distributed across large areas, and by temporal heterogeneity, because different stands are harvested and renewed at different times, creating a mosaic of stands in distinct growth stages [10]. The same stand can rapidly evolve from a recently intervened area with sparse vegetation cover to a forest-like structure in the pre-harvesting phase, and these changes occur within a relatively short production cycle. This dynamic mosaic can strongly influence wildlife presence and behavior across plantation landscapes [8].

Previous studies in Europe have shown that Eucalyptus plantations can influence the spatial and temporal ecology of several taxa, including mesocarnivores, small mammals, and wild ungulates [8,9,11,12,13,14,15]. However, wildlife responses to production forests are unlikely to depend only on whether plantations are present. Instead, they may vary with stand age, harvesting phase, retained vegetation, edge structure, and the intensity of recent management interventions [10,16,17]. For wild ungulates, this distinction is especially important because forestry operations may temporarily reduce cover and increase disturbance, while later vegetation recovery may restore forage and refuge conditions [18]. This suggests that Eucalyptus plantations should not be treated as ecologically uniform systems and that management-related disturbance may be an important driver of wildlife responses.

Portugal holds the largest area of exotic planted *Eucalyptus* spp. in Europe, with *Eucalyptus globulus* representing 26.2% of the country’s forest area, approximately 845,000 hectares, predominantly located in the northern and central regions [19]. Although mainland Portugal includes a diversity of forest and semi-natural formations, including pine forests, evergreen and deciduous native broadleaf systems, shrublands, pastures, and agricultural areas, the national forest inventory identifies eucalypt stands as one of the major forest formations in mainland Portugal [19], reinforcing their relevance as a study system. This species is produced primarily for the pulp and paper industry [5,20]. Nevertheless, the specific effects of stand-level management practices within these plantations on wildlife occupancy and behavior remain largely underexplored [9,17,21]. While several studies have examined broader wildlife responses to Eucalyptus plantations (e.g., [16]), fewer have focused on how different management practices influence species occupancy, and information on wild ungulates remains particularly scarce [11,12].

This gap is particularly relevant for roe deer (*Capreolus capreolus*). Roe deer are among the most widespread ungulates in Europe [22] and have expanded their range and abundance in mainland Portugal in recent decades [23]. Because this expansion is linked to land-cover change and occurs near the southwestern edge of the species’ European distribution, Portuguese roe deer populations provide a useful case for examining how adaptable ungulates use human-modified Mediterranean landscapes [23,24,25]. Roe deer use depends on the balance between vegetation cover, forage availability, landscape composition, forest structure, and disturbance risk [26,27,28], and the species may influence forest ecosystems through browsing effects on vegetation structure and regeneration [29,30]. These characteristics make roe deer an appropriate focal species for assessing how production-forest management affects medium-sized ungulate site use [31].

In the context of expanding Eucalyptus plantations in some regions and the increasing range and abundance of roe deer populations, it is important to understand how exotic forest management shapes roe deer occupancy/site use (ψ) and detectability (*p*). Previous work using the same camera-trap dataset has examined broader environmental and landscape-level drivers of wild ungulate responses to Eucalyptus plantations [8]. Therefore, the present study deliberately focuses on the management dimension of these production forests, rather than repeating a broader habitat-selection analysis with a subset of the same data. Specifically, we investigated whether stand-level variables directly linked to Eucalyptus plantation management explain part of the variation in roe deer responses within managed plantation areas. Using camera-trap data, we evaluated time since intervention and production regimen (afforestation, coppice, or reforestation) as the main ecological predictors of roe deer site use because they represent the temporal and structural dimensions of silvicultural disturbance. We also considered production status as an additional stand-level covariate because wildfire- or disease-related damage can abruptly alter stand structure and trigger further intervention, potentially affecting roe deer responses. Finally, we evaluated stand area because larger stands may alter the effective sampling coverage of individual camera-trap stations and may also reflect broader disturbance conditions within the plantation mosaic (Table 1).

We expected the ecological effect of time since intervention to depend on the production regimen because each regimen represents a different disturbance trajectory rather than simply a different plantation label. Afforestation establishes a new Eucalyptus stand but does not involve the removal of a previously mature Eucalyptus structure. Coppice represents an intermediate disturbance because trees resprout after harvest without full soil preparation. Reforestation, by contrast, is expected to generate the strongest short-term disturbance because it typically involves stump removal, soil tillage, replanting, heavier machinery, and a stronger reset of stand structure. Because roe deer use is influenced by vegetation cover, forage availability, and avoidance of acute disturbance, the recovery of site use after intervention should not be uniform across regimens [26,27,28,32].

Accordingly, we tested three a priori hypotheses: H1, production regimens differ in their effects on roe deer site use because they represent distinct levels of habitat disturbance and structural alteration; H2, time since intervention is positively associated with roe deer site use as vegetation structure recovers and disturbance intensity declines after management interventions; and H3, the effect of time since intervention differs among production regimens, with the strongest positive recovery expected in reforestation stands because these experience the most severe short-term disturbance.

## 2. Materials and Methods

### 2.1. Study Area

**Figure 1:**
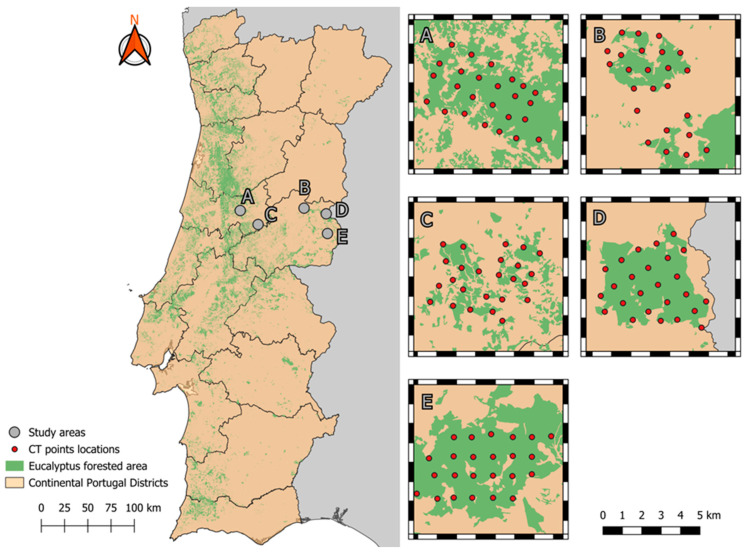
Selected study areas (grey points: A—Góis; B—Fundão; C—Pampilhosa; D—Penamacor; E—Penha Garcia) dominated by Eucalyptus plantations (green area) in Continental Portugal. The red dots represent the camera traps (CT) deployed in the study areas during one of the sampling periods.

Our study was conducted in central Portugal and included five areas dominated by exotic Eucalyptus plantations: Góis (40.20° N, 8.15° W), Fundão (40.21° N, 7.33° W), Pampilhosa (40.07° N, 7.93° W), Penamacor (40.17° N, 7.04° W), and Penha Garcia (39.97° N, 7.04° W) (Figure 1). This region is characterized by hot, dry summers and cold, wet winters, with a mean annual temperature of 16 °C and a mean annual rainfall of 750 mm (data from the Portuguese Institute for Sea and Atmosphere—IPMA—collected from 1971 to 2000). Besides the Eucalyptus plantations, the region’s landscape includes some *Pinus* spp. plantations, native woodlands (e.g., *Quercus suber* L. and *Quercus robur* L.), and Mediterranean shrubland patches composed, among other species, of *Cistus ladanifer* L. and *Arbutus unedo* L.

All the plantations studied are privately managed by the same company and follow the Forest Stewardship Council (FSC) sustainability standards. Each of the five study areas was located at least 10 km from each other to ensure spatial sampling independence and was large enough to encompass the 16 km^2^ sampling grid (see the Sampling Design section). These Eucalyptus plantations are aimed at wood production for the paper industry. Although stands are spatially homogeneous (i.e., only *Eucalyptus* spp. trees are present), the plantations exhibit a temporal heterogeneity, i.e., the plantation structure varied along the production cycle. Due to this structural variation throughout the production cycle, each study area comprised stands with distinct ages and development stages.

The production cycles are classified into three production regimens: afforestation, coppice, and reforestation (see Eucalyptus stand production regimen variable in Table 1 for details), but stochastic events, such as wildfires or phytopathological outbreaks, can interrupt this cycle. In such a context, the affected trees are cut down prematurely to harvest the burned wood or to prevent the disease from spreading, respectively. Such premature harvesting allows the beginning of a new production cycle.

### 2.2. Field Study Design

In each study area, we set up 25 camera traps (Cuddeback 20 Megapixel IR H-1453 white series; Cuddeback/Non Typical, Inc., De Pere, WI, USA) in a 1 km grid (Figure 1) to reduce short-distance dependence between neighboring stations and to ensure broad coverage of each plantation landscape [33]. Although the intended sampling design followed a 1 km grid, exact spacing could not always be maintained because camera placement had to be adjusted to local terrain, access constraints, stand boundaries, and suitable deployment points. Therefore, some camera traps were positioned at distances shorter than 1 km; these sites were not excluded from the analysis, but spatial non-independence was subsequently evaluated through residual spatial autocorrelation tests and, where necessary, corrected by including spatial coordinates in the site-use component of the model. Because roe deer are mobile and camera traps sample only a restricted portion of each stand, this spacing should be viewed as a design choice to reduce, rather than guarantee, spatial independence. Cameras were active 24 h a day for 30 consecutive days (30 trap nights) in each survey period. We set up four survey periods: February–May 2019, June–September 2019, January–May 2020, and July–September 2020, representing the wet and dry seasons of each year. Camera traps were not baited, placed 40 to 60 cm above ground, and recorded three photographs per detection event, with a minimum 30 s interval of inactivity before a new trigger.

### 2.3. Detection and Occupancy Candidate Variables

To test whether stand-level plantation management explained part of the variation in roe deer site-use and detection patterns, we used variables directly linked with the Eucalyptus management scheme: time since intervention, production status, stand size, and production regimen (see Table S1 for summary statistics and counts for these variables). These variables were selected to address the management-focused scope of this study and should therefore be interpreted as management-linked predictors rather than as a complete set of environmental drivers of roe deer space use. For a detailed rationale and description of each variable, see Table 1.

### 2.4. Statistical Analysis

#### 2.4.1. Data Handling

We only included independent detections in the analysis, i.e., those separated by at least 30 min from a previous detection of the same species in the same camera. The detections of roe deer were grouped into 5-day occasions. We registered whether the target species was detected (1) or not (0) for each occasion. Data handling was conducted using R Statistical Software (R version 4.4.2) [34] with the package “camtrapR” (Version 2.3.0) [35] and “unmarked” (Version 1.5.1) [36,37].

#### 2.4.2. Occupancy Modeling

Before model building, we standardized the continuous covariates using global centering and scaling across the full dataset so that effect sizes remained comparable among sessions. We screened collinearity separately for the base detection covariates and the base site-use covariates by estimating Variance Inflation Factors (VIF), retaining predictors with VIF < 5 [38]. Squared terms were not included in this generic screen because they were later evaluated as structured polynomial extensions of the retained linear predictors.

We analyzed the data as four independent single-season models, one for each sampling session, because closure is much more plausible within a session than across seasons and years and because our sampling design was not suited to estimate colonization–extinction dynamics due to the locations and number of functional camera-traps changing between seasons (see Table S2). We used the occupancy framework to model detection probability (*p*) and occupancy/site-use probability (ψ) [39], but for ecological interpretation we treat ψ cautiously as probability of site use during the sampling session rather than permanent occupancy of a bounded stand, given the mobility of roe deer and the fine spatial grain of camera-trap sampling [40].

Within each session, model selection proceeded in five steps: (1) selection of the baseline detection structure, (2) selection of ecological candidate models for site use, (3) evaluation of the quadratic effect of predictors (test for non-linearity), (4) testing and correction of residual spatial autocorrelation, and (5) validation of the final retained model, including goodness-of-fit (GOF), overdispersion, and QAICc sensitivity analysis.

We first selected the baseline detection structure by fitting candidate detection models while keeping the site-use component constant (ψ ~ 1). Detection candidates were *p* ~ 1, *p* ~ Stand_Size, *p* ~ Stand_Status, and *p* ~ Stand_Size + Stand_Status. The site-use candidate set included ψ ~ 1, single-predictor models, additive combinations of time since intervention, production regimen, and production status, and the interaction ψ ~ Regime * T_Intervention, which represented our a priori hypothesis that recovery after disturbance would differ among regimens. Production status was considered to be a candidate covariate for both detection and site use because wildfire- or disease-related damage may alter stand structure, human intervention intensity, and short-term habitat suitability.

After selecting the best linear detection structure and the best linear site-use structure, we evaluated polynomial extensions only for the retained continuous predictors. Specifically, we compared linear *p* ~ Stand_Size versus *p* ~ Stand_Size + Stand_Size^2^ in the detection component, and linear ψ ~ T_Intervention versus ψ ~ T_Intervention + T_Intervention^2^ in the retained state component. Quadratic terms were retained only when the polynomial extension improved the Akaike Information Criterion for small sample sizes (AICc) by at least 2 units [41].

Residual spatial autocorrelation was assessed separately in each session using Moran’s I applied to site-level residuals from the best non-spatial model, and was considered relevant when Moran’s I was statistically significant. When spatial structure was detected, scaled UTM X and Y coordinates were added to the site-use component of the retained non-spatial candidate structure, and the corrected version was then re-evaluated. We retained the spatial correction only when it reduced residual Moran’s I to a non-significant level. This step was included because camera-trap occupancy analyses can retain spatial dependence even when field spacing is designed to reduce it [42].

Only the final retained model in each session was taken forward to validation. We evaluated observed-versus-predicted calibration using Spearman rank correlation between site-use and detection estimates, MacKenzie–Bailey bootstrap goodness-of-fit, and the overdispersion parameter (ĉ) using 500 simulations per final model [43]. For sessions showing evidence of overdispersion in the MacKenzie–Bailey bootstrap GOF assessment, we estimated ĉ and re-ranked the relevant candidate set with QAICc as a sensitivity analysis rather than as the primary model-selection criterion [44]. After session-specific model selection and validation, we summarized the retained detection and site-use structures across the four sessions to identify recurring patterns and differences in support among periods.

Data analysis was conducted in R Statistical Software (R version 4.4.2) [34] using the packages “unmarked” (Version 1.5.1) [36,37], “AICcmodavg” (Version 2.3.2) [45], and “MuMIn” (Version 1.47.5) [46].

## 3. Results

In total, 498 camera traps (CT) were placed in all study areas (Fundão—98 CT; Penamacor—100 CT; Pampilhosa—100 CT; Góis—100 CT; and Penha Garcia—100 CT). However, in some study areas, the number of functional cameras decreased due to mechanical issues or theft. Additionally, of the 480 functional cameras, 401 were set inside the company’s owned/managed areas, for which we had management information. Therefore, after removing the cameras that were not placed on Eucalyptus stands within the entire survey period from our dataset, we collected data from 375 fully operational cameras in the target areas of this study during the four sampling periods (referred to as sessions). Roe deer were detected on 341 of 2214 5-day occasions across all monitoring periods and all operational cameras, corresponding to approximately 15% of the sampling occasions retained for analysis. The distribution of retained camera-trap sites among sessions and study areas is summarized in Table S2.

### 3.1. Summary of Retained Models Across Sessions

Across sessions, the stand size was retained primarily in the detection component, whereas the site-use structure varied among periods. The 2019 wet-season session used a constant site-use model with a quadratic effect of stand size on detection. The 2019 dry-season session retained quadratic effects of stand size on detection and time since intervention on site use but was weakened by strong overdispersion and QAICc sensitivity. The 2020 wet-season session retained a positive effect of time since intervention after accounting for broad spatial structure, whereas the 2020 dry-season session retained a regimen × time since intervention interaction and provided the strongest overall support for disturbance-related differences in site use and regimen-specific temporal responses. Production status was retained in the detection component of both 2020 sessions, suggesting that damaged versus in-production stands contributed to detectability differences, although this variable did not emerge as a consistent driver of site use in the final ecological models. Parameter estimates for the final retained models are provided in Table 2, whereas Table 3 summarizes the corresponding model-selection and validation metrics, including Spearman rank correlation, residual Moran’s I, overdispersion (ĉ), goodness-of-fit, and QAICc sensitivity.

### 3.2. Session-Specific Model Results

In the 2019 wet-season session, the best retained model was ψ ~ 1 and *p* ~ Stand_Size + Stand_Size^2^. Detection was highest at intermediate stand sizes and declined in the largest stands, but no ecological site-use covariate outperformed a constant site-use model (ψ ~ 1). Residual spatial autocorrelation remained significant after testing spatial correction, so this session is informative mainly for detectability and should be interpreted cautiously for site-use inference. This interpretation is supported by the validation metrics in Table 3, which show residual Moran’s I close to zero significance, moderate overdispersion (ĉ = 1.42), and a significant GOF result (*p* = 0.038). The fitted detection pattern is shown in Figure S1 and reinforces that this session mainly informs detectability rather than site use, given that camera-trap sampling coverage can vary with the effective area sampled within larger stands [40].

In the 2019 dry-season session, the retained model was ψ ~ T_Intervention + T_Intervention^2^ and *p* ~ Stand_Size + Stand_Size^2^. Both relationships were hump-shaped, suggesting the highest fitted site use and detectability at intermediate values. However, this session also showed the strongest lack of fit and overdispersion, and QAICc sensitivity shifted the top ecological model for site use from time since intervention to the constant model (ψ ~ 1). As shown in Table 3, this session had the highest overdispersion estimate (ĉ = 3.45) and the lowest GOF support (*p* = 0.004), reinforcing that the apparent site-use process signal should be treated as unstable. Accordingly, the site-use process signal for this session should be considered suggestive rather than definitive. The fitted stand size and time since intervention curves are shown in Figures S2 and S3.

In the 2020 wet-season session, the retained model was ψ ~ T_Intervention + X + Y and *p* ~ Stand_Size + Stand_Status. This indicates that detectability variation was influenced not only by stand size but also by whether stands were in production or damaged. Site use increased with time since intervention, but the model also retained strong spatial terms, indicating that an unmeasured broad-scale spatial gradient remained relevant. After including coordinates, residual spatial autocorrelation was reduced to a borderline/non-significant level, making this one of the more informative sessions. Table 3 shows that residual Moran’s I was reduced to a borderline value (*p* = 0.054), although overdispersion remained above 1 (ĉ = 2.03), indicating improved but still imperfect model fit. The corresponding parameter estimates are reported in Table 2.

**Figure 2:**
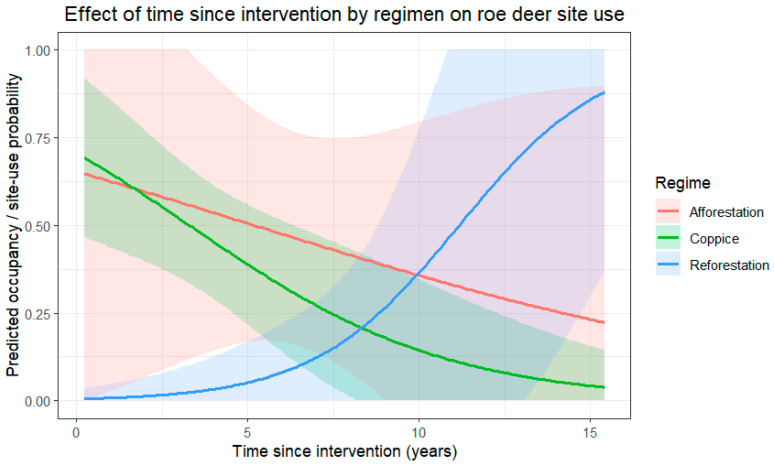
Predicted roe deer site-use probability along the gradient of time since intervention for each Eucalyptus production regimen (afforestation, coppice, and reforestation) in the dry season of the 2020 session. Predictions are based on the final retained occupancy/site-use model, and shaded areas indicate 95% confidence intervals.

In the 2020 dry-season session, the retained model was ψ ~ Regime × T_Intervention and *p* ~ Stand_Size + Stand_Status. As in the 2020 wet-season session, production status contributed to the retained detection structure rather than to the final site-use component. This was the strongest-supported session: residual spatial autocorrelation was not detected, GOF was adequate, and overdispersion was not evident. Consistent with this interpretation, Table 3 shows non-significant residual Moran’s I (*p* = 0.398), no evidence of overdispersion (ĉ = 0.91), and adequate goodness-of-fit (*p* = 0.502). Reforestation stands showed lower site use than afforestation stands at the reference time since intervention, and the interaction structure indicated that temporal site-use trajectories differed among regimens (Figure 2). The corresponding parameter estimates are also reported in Table 2. Observed-versus-predicted detection and site-use relationships for the final retained models across all four sessions are shown in Figure S4.

## 4. Discussion

The effects of management options on roe deer site use vary between years and seasons. Reforestation appears to be the most disruptive management regimen for roe deer site use. Operationally, reforestation generally follows afforestation and subsequent coppice cycles and usually involves stump removal, soil preparation, heavier machinery, and a stronger structural reset of the stand. These interventions are likely to reduce shelter availability and increase acute disturbance, conditions that roe deer are known to avoid [27,32]. Our strongest session-level result indicated lower roe deer site-use probability in reforestation stands than in afforestation stands, reinforcing the view that it is not the mere presence of Eucalyptus plantations that shapes roe deer responses, but rather the intensity and timing of silvicultural disturbance within them. This supports H1 and confirms that roe deer responses are influenced not just by the presence of Eucalyptus plantations, but also by the management practices implemented within these human-modified environments. These findings are consistent with previous evidence that ungulates may alter their spatial and temporal behaviour within Eucalyptus plantation landscapes [8]. They are also compatible with broader roe deer habitat-use studies showing that forest structure, cover availability, forage resources, and disturbance can influence how roe deer use managed forest systems [28,47,48]. Thus, our results should not be interpreted as a general test of whether roe deer prefer Eucalyptus plantations over other habitats, but rather as evidence that management phase and post-intervention recovery can shape roe deer site use within Eucalyptus production stands.

The effects of time since intervention further support this interpretation, although this support came mainly from the 2020 analyses. In our models, roe deer use did not respond uniformly to time since intervention across all sessions or regimens. This effect was approximately linear in the 2020 wet season and interaction-based in the 2020 dry season, whereas the 2019 dry season suggested a possible non-linear response peaking at intermediate times, though that pattern should be interpreted cautiously given the weaker model support, strong overdispersion, and QAICc sensitivity. Ecologically, these results suggest that roe deer may re-use plantation stands after disturbance, but the clearest support for this interpretation came from the 2020 sessions and, in the 2020 dry season, mainly from reforestation stands rather than uniformly across all regimens. As cover and foraging opportunities are restored, some stands may again become usable by this adaptable cervid. This is plausible given that roe deer are selective feeders [47] and are known to exploit human-modified habitats when disturbance is not continuous and sufficient structural refuge is available [49,50]. These results are broadly consistent with H2, which predicted that site use would increase with time since intervention as vegetation structure recovers and disturbance intensity declines. However, because the strength and functional form of this relationship differed among sessions, support for H2 should be regarded as moderate. This interpretation is also compatible with evidence that terrestrial mammal assemblages in Eucalyptus plantations vary with stand age, reinforcing the idea that recovery after intervention can alter habitat use through time [16].

More broadly, these patterns suggest that roe deer responses to plantation management may depend not only on disturbance intensity but also on how different silvicultural contexts change through time. Because different stands are often at different points in the management cycle, Eucalyptus plantations can form a shifting mosaic of conditions, ranging from recently disturbed reforestation areas to comparatively less disruptive afforestation or coppice stands and older recovering stands. Such heterogeneity may allow roe deer to move among stands and exploit resources that vary in availability through time, although this mechanism was not directly tested here. In this sense, plantation landscapes may function as a form of temporal habitat complementation [51], in which different stands supply different resources or conditions at different times. However, this interpretation should not obscure the fact that reforestation itself appears to represent a period of particularly low suitability. If reforestation occurs over large contiguous areas, or if many stands enter this regimen simultaneously, the resulting disturbance could plausibly reduce habitat continuity, constrain movements, and create temporary barriers for roe deer, with possible consequences for dispersal and persistence [52]. However, this landscape-scale mechanism was not directly tested here and should be evaluated with spatially explicit disturbance and movement data. Our findings offer limited support for H3, suggesting that the effect of time since intervention varies across regimens. While not statistically significant over time, this pattern was observed in the 2020 dry season, in which the interaction between regimen and time since intervention indicated distinct temporal site-use responses across the three silvicultural contexts. Unlike the other regimen, the reforestation regimen showed a positive trend in site-use probability over time (Table 2), with a notable difference in roe deer presence during the first five years after intervention (Figure 2). These shifting responses align with previous evidence that ungulate spatiotemporal behavior changes across plantation phases within Eucalyptus landscapes [8].

A secondary but important result concerns stand size, which, in our analysis, was retained in the detection component, and in two sessions, its effect was better described as non-linear. This means that larger stands should not automatically be interpreted as poorer habitat for roe deer. A single camera trap is able to sample only a small portion of a large stand, so detectability may decline even if roe deer continue to use the area [40]. For that reason, management recommendations derived from this study should not be based on stand-size effects alone unless future work demonstrates a consistent relationship with the site-use process at broader spatial scales (e.g., by using a small-scale sampling grid). Still, at the landscape level, very large reforestation blocks could plausibly amplify disturbance intensity and reduce short-term habitat suitability, a hypothesis that deserves direct testing with spatially explicit disturbance metrics.

Several limitations of this study should be acknowledged explicitly. First, our models focused on stand-level management variables and did not reintroduce the broader environmental and landscape-level predictors previously evaluated using the same camera-trap dataset [8]. Therefore, our results should be interpreted as a management-focused complement to that broader habitat-selection analysis, rather than as a complete model of all ecological drivers of roe deer site use. Second, because roe deer are mobile and camera traps sample only a restricted temporal window, the parameter ψ is better interpreted here as a probability of site use during each sampling session rather than permanent occupancy of a bounded stand. Third, some sessions retained residual spatial structure or overdispersion, indicating that inference strength differed among periods and that unmeasured heterogeneity remains. Even with these limitations, the strongest-supported models indicate that disturbance intensity and post-intervention recovery are more informative for management interpretation than stand size alone. Future studies integrating landscape context and movement data, including GPS-based tracking, will be important for testing these management implications more directly.

## 5. Conclusions

From a management perspective, our results support the cautious implication that reducing the spatial footprint and contiguity of intensive reforestation operations may help maintain greater age- and disturbance-related heterogeneity within plantation mosaics. In parallel, maintaining less disturbed areas within plantation landscapes may be a useful precautionary measure, although their role as refuges or movement stepping stones for roe deer should be tested directly in future studies using landscape context and movement data. Such measures are more closely aligned with the disturbance signal identified in the strongest models than broad structural prescriptions based only on stand area.

To summarize, our study shows that Eucalyptus plantations should not be treated as ecologically uniform systems. Their effects on roe deer depend on when and how stands are managed, and on how disturbance is distributed across the landscape. Integrating wildlife conservation into plantation planning, particularly by limiting the extent, synchrony, and intensity of disruptive interventions, may improve compatibility between forestry production and biodiversity conservation. Future studies incorporating landscape context and direct movement data, such as GPS-based tracking, will be essential for clarifying the mechanisms underlying plantation use and for translating these session-specific patterns into broader and more robust management guidance.

## Figures and Tables

**Table 1:** Variables used to perform the detection and occupancy models of roe deer. Columns contain the variables’ names, acronyms, descriptions, rationale, and predictions.

Variables (Acronym)	Variable Description	Rationale and Predictions
Eucalyptus stand’s production status (Stand_Status)	This categorical variable indicates whether the Eucalyptus stand’s production was normal or damaged by either wildfire or phytological pathology. Both occurrences stop or hinder the normal development of the Eucalyptus trees in that stand. Wildfire directly destroys the growing trees, while disease affects their normal growth and can spread to other nearby stands. Therefore, mechanical interventions are necessary to clear the affected area and apply protective measures for the surrounding trees.	When Eucalyptus production is damaged, subsequent mechanical intervention and sudden environmental changes may create disturbance and alter stand structure, potentially influencing both roe deer site use and camera-trap detectability. We expected roe deer site use to be lower in damaged-production stands if recent disturbance reduced habitat suitability or increased perceived risk. However, because wildfire, disease, and associated interventions can also modify vegetation density and visibility, production status was also considered as a candidate detection covariate.
Eucalyptus stand size in hectares (Stand_Size)	This numerical variable measures the total area of each Eucalyptus stand in hectares.	Stand size was considered primarily as a detection-related covariate, because a single camera trap samples a smaller proportion of a larger stand, potentially reducing detection probability even when roe deer use the stand. Stand size may also indirectly reflect the spatial extent of management interventions within the plantation mosaic, but any ecological interpretation of stand-size effects on site use was treated cautiously.
Eucalyptus stand production regimen (Regime)	This categorical variable defines the Eucalyptus stand’s production regimen, which includes afforestation, coppice, and reforestation. Afforestation represents the first cycle of plantation of Eucalyptus trees in the stand where there was no previous Eucalyptus. Coppice is a regimen that allows the trees to resprout to establish a new growth rotation after clear-cutting the stand to harvest the developed trunks. This regimen is only allowed twice before the reforestation regimen follows. The reforestation regimen happens when the Eucalyptus stumps are removed via mechanical force, and new saplings are transplanted into the stand. This reforestation is permitted only once, except for unexpected cycle-ending interventions, such as wildfire or disease. The coppice regimen is also allowed twice after the reforestation regimen. Afterward, the plantation of Eucalyptus stops at that stand.	Production regimens represent different management trajectories and disturbance intensities. We expected roe deer site use to vary among regimens because afforestation, coppice, and reforestation differ in the degree of structural reset, soil disturbance, machinery use, and post-intervention vegetation development. Reforestation was expected to have the strongest negative short-term association with site use because it involves removing previously established Eucalyptus trees and stumps, preparing the soil, and replanting, thereby resetting stand structure to an early stage with reduced cover and structural complexity. Coppice was expected to represent an intermediate disturbance because resprouting occurs after harvest without full soil preparation, whereas afforestation was treated as the reference condition for evaluating regimen-specific differences.
Time since intervention (T_Intervention)	This numerical variable measures the stand age, which is the time since the previous intervention on the Eucalyptus stand in days, which was later converted to years for the models. The interventions are considered a change in regimen that involves human presence, manual labor, heavy machinery, and environmental disturbance on the stand.	Time since intervention was used as a proxy for post-management recovery. If recent interventions reduce habitat suitability through disturbance, vegetation removal, or reduced cover, we expected roe deer site use to increase as the time since intervention increased and the stand structure partially recovered. This effect was expected to differ among production regimens because the intensity of the initial disturbance and the trajectory of vegetation recovery are not equivalent across afforestation, coppice, and reforestation.

**Table 2:** Parameter estimates for the final retained models of occupancy/site use (ψ) and detection probability (*p*) for each sampling session. Estimates include coefficient values, standard errors (SE), z values, and *p*-values.

Session	Process	Term	Estimate	SE	z	*p*-Value
Sess1_Wet_2019	Occupancy (ψ)	(Intercept)	0.793	0.334	2.380	1.7500 × 10^−2^
	Detection (*p*)	(Intercept)	−0.924	0.221	−4.180	2.9700 × 10^−5^
		Stand_Size	−1.693	0.877	−1.930	5.3600 × 10^−2^
		Stand_Size^2^	−2.420	1.368	−1.770	7.6900 × 10^−2^
Sess2_Dry_2019	Occupancy (ψ)	(Intercept)	0.574	0.582	0.985	3.2440 × 10^−1^
		T_Intervention	−1.011	0.512	−1.974	4.8400 × 10^−2^
		T_Intervention^2^	−1.628	0.832	−1.957	5.0400 × 10^−2^
	Detection (*p*)	(Intercept)	−1.270	0.352	−3.610	3.0600 × 10^−4^
		Stand_Size	−1.650	1.562	−1.060	2.8999 × 10^−1^
		Stand_Size^2^	−2.090	1.974	−1.060	2.8940 × 10^−1^
Sess3_Wet_2020	Occupancy (ψ)	(Intercept)	0.201	0.334	0.603	5.4700 × 10^−1^
		T_Intervention	0.771	0.355	2.172	2.9800 × 10^−2^
		X_SpatialCorrection	1.536	0.380	4.040	5.3500 × 10^−5^
		Y_SpatialCorrection	1.392	0.363	3.832	1.2700 × 10^−4^
	Detection (*p*)	(Intercept)	0.509	0.621	0.820	4.1250 × 10^−1^
		Stand_Size	−0.415	0.213	−1.950	5.1500 × 10^−2^
		Stand_StatusForestry_Production	−1.070	0.630	−1.700	8.9400 × 10^−2^
Sess4_Dry_2020	Occupancy (ψ)	(Intercept)	−0.040	0.652	−0.061	9.5110 × 10^−1^
		RegimeCoppice	−0.549	0.764	−0.719	4.7240 × 10^−1^
		RegimeReforestation	−2.631	1.262	−2.085	3.7100 × 10^−2^
		T_Intervention	−0.479	0.823	−0.582	5.6030 × 10^−1^
		RegimeCoppice:T_Intervention	−0.565	0.954	−0.593	5.5350 × 10^−1^
		RegimeReforestation:T_Intervention	2.329	1.521	1.532	1.2560 × 10^−1^
	Detection (*p*)	(Intercept)	−2.380	1.214	−1.960	4.9480 × 10^−2^
		Stand_Size	−1.010	0.363	−2.770	5.6200 × 10^−3^
		Stand_StatusForestry_Production	1.890	1.213	1.550	1.2020 × 10^−1^

**Table 3:** Summary of model selection and validation results for each sampling session, including the top-ranked model by AICc, Spearman rank correlation between occupancy/site-use and detection estimates, residual Moran’s I, overdispersion parameter (ĉ), goodness-of-fit (GOF) *p*-value, and the top-ranked model by QAICc.

Session	Top Model by AICc	Spearman rho	Residual Moran’s I	ĉ	GOF (*p*-Value)	Top Model by QAICc
Sess1_Wet_2019	ψ ~ 1; *p* ~ Stand_Size + Stand_Size^2^	0.151	4.864 × 10^−9^	1.4215	0.0380	ψ ~ 1; *p* ~ Stand_Size + Stand_Size^2^
Sess2_Dry_2019	ψ ~ T_Intervention + T_Intervention^2^; *p* ~ Stand_Size + Stand_Size^2^	0.388	6.940 × 10^−1^	3.4473	0.0040	ψ ~ 1; *p* ~ Stand_Size + Stand_Size^2^
Sess3_Wet_2020	ψ ~ T_Intervention + X_SC + Y_SC; *p* ~ Stand_Size + Stand_Status	0.539	5.413 × 10^−2^	2.0276	0.0080	ψ ~ T_Intervention + X_SC + Y_SC; *p* ~ Stand_Size + Stand_Status
Sess4_Dry_2020	ψ ~ Regime * T_Intervention; *p* ~ Stand_Size + Stand_Status	0.465	3.982 × 10^−1^	0.9090	0.5020	

**Note:** In model formulas, * denotes an interaction term rather than multiplication. Thus, Regime * T_Intervention includes the main effects of Regime and T_Intervention, plus their interaction.

## Data Availability

The data and R code supporting the findings of this study are available from the corresponding author upon reasonable request.

## Supplementary Materials

The following supporting information can be downloaded at: https://www.mdpi.com/article/10.3390/ani16111613/s1, Figure S1: Predicted detection probability of roe deer as a function of Eucalyptus stand size (ha) in the wet season of the 2019 session. The solid line represents the fitted relationship from the final retained detection model, and the shaded area indicates the 95% confidence interval; Figure S2: Predicted detection probability of roe deer as a function of Eucalyptus stand size (ha) in the dry season of the 2019 session. The solid line represents the fitted relationship from the final retained detection model, and the shaded area indicates the 95% confidence interval; Figure S3: Predicted roe deer site-use probability as a function of time since intervention in the dry season of the 2019 session. The solid line represents the fitted relationship from the final retained occupancy/site-use model, and the shaded area indicates the 95% confidence interval; Figure S4: Validation plots for the final retained roe deer occupancy/site-use models, showing observed versus predicted site-level detection rates across the four sampling sessions. Solid lines show the fitted linear relationship between observed and predicted values, and dashed lines indicate the 1:1 line of perfect agreement; Table S1: Summary statistics and counts for categorical and numerical variables: stand’s production status, stand’s area in hectares, stand’s regime, and time since last intervention in days; Table S2: Number of camera-trap sites retained in each sampling session and corresponding distribution among the five study areas considered in the occupancy analyses.

## Abbreviations

The following abbreviations are used in this manuscript:

AICc	Akaike Information Criterion corrected for small sample sizes
CT	Camera trap(s)
FSC	Forest Stewardship Council
GOF	Goodness-of-fit
IPMA	Portuguese Institute for Sea and Atmosphere
QAICc	Quasi-Akaike Information Criterion corrected for small sample sizes
SE	Standard error
UTM	Universal Transverse Mercator
VIF	Variance Inflation Factor(s)
*p*	Detection probability
ψ	Probability of site use
