# Supplementary material for: Eucalyptus Plantation Management Shapes Roe Deer Site-Use Patterns
Source: Animals (Basel). 2026 May 26;16(11):1613. doi: 10.3390/ani16111613 (PMC13255817; doi:10.3390/ani16111613)
Supplement: Supplementary file 1 [file animals-16-01613-s001.zip › Figure S1.pdf]

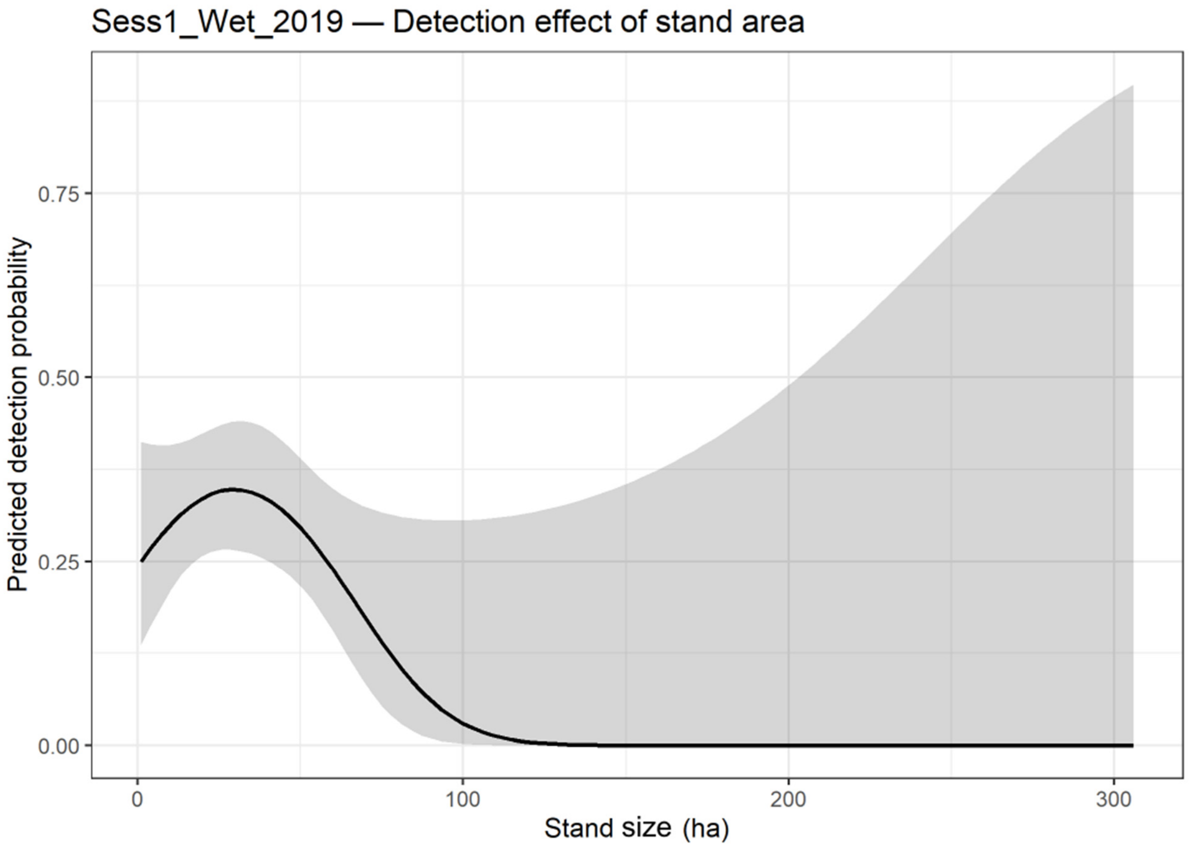

Figure S1. Predicted detection probability of roe deer as a function of Eucalyptus stand size (ha) in the wet season of the 2019 session. The solid line represents the fitted relationship from the final retained detection model, and the shaded area indicates the 95% confidence interval.
