# Supplementary material for: Eucalyptus Plantation Management Shapes Roe Deer Site-Use Patterns
Source: Animals (Basel). 2026 May 26;16(11):1613. doi: 10.3390/ani16111613 (PMC13255817; doi:10.3390/ani16111613)
Supplement: Supplementary file 1 [file animals-16-01613-s001.zip › Figure S3.pdf]

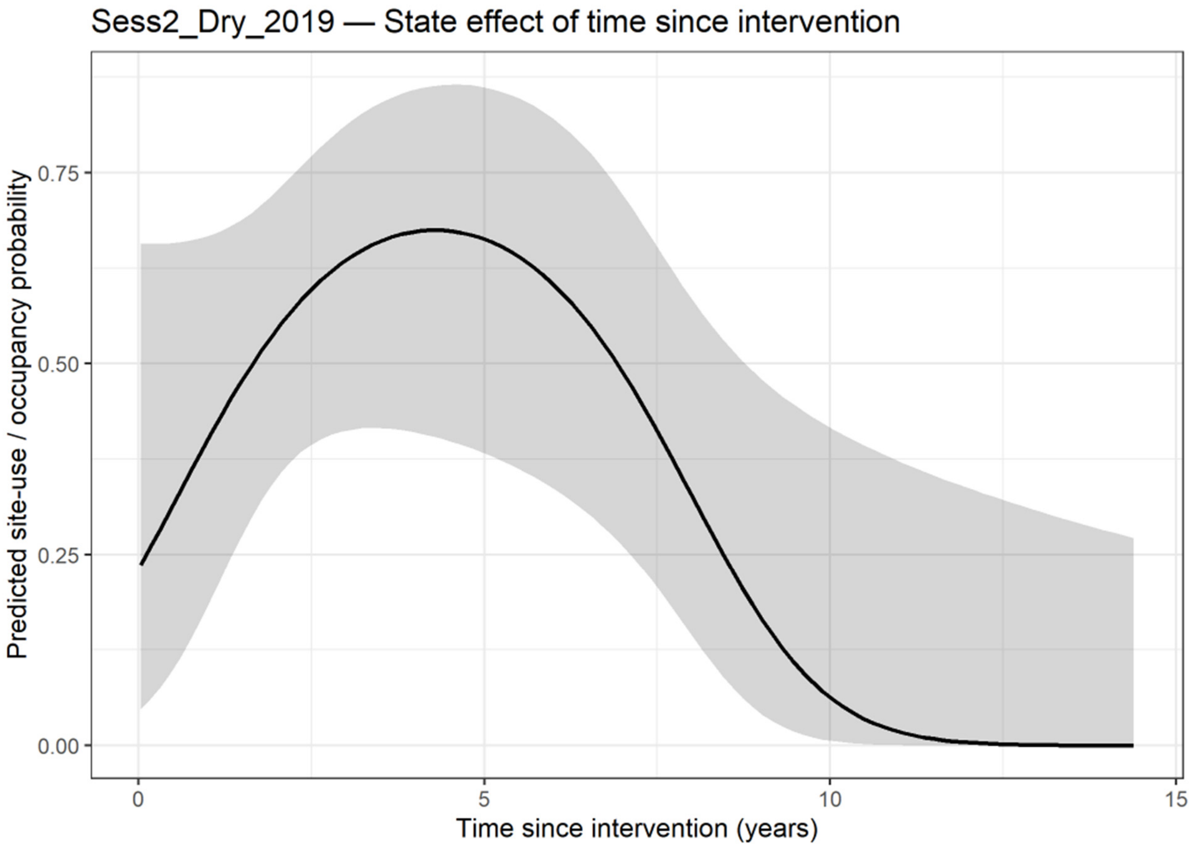

Figure S3. Predicted roe deer site-use probability as a function of time since intervention in the dry season of the 2019 session. The solid line represents the fitted relationship from the final retained occupancy/site-use model, and the shaded area indicates the 95% confidence interval.
