# Supplementary material for: Eucalyptus Plantation Management Shapes Roe Deer Site-Use Patterns
Source: Animals (Basel). 2026 May 26;16(11):1613. doi: 10.3390/ani16111613 (PMC13255817; doi:10.3390/ani16111613)
Supplement: Supplementary file 1 [file animals-16-01613-s001.zip › Figure S4.pdf]

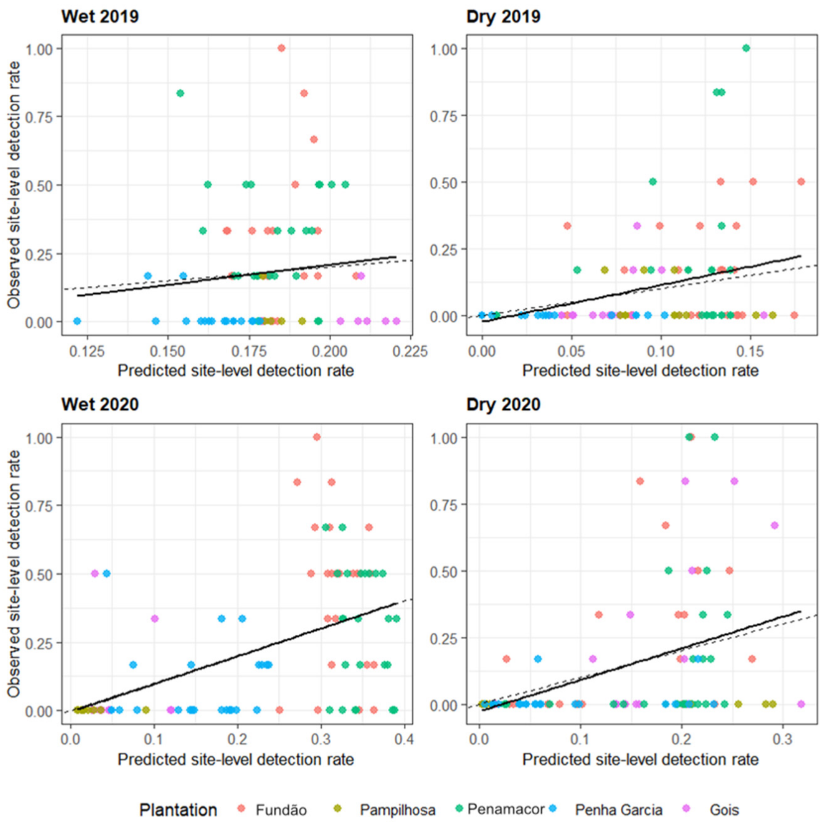

Figure S4. Validation plots for the final retained roe deer occupancy/site-use models, showing observed versus predicted site-level detection rates across the four sampling sessions. Solid lines show the fitted linear relationship between observed and predicted values, and dashed lines indicate the 1:1 line of perfect agreement.
