# Supplementary material for: Eucalyptus Plantation Management Shapes Roe Deer Site-Use Patterns
Source: Animals (Basel). 2026 May 26;16(11):1613. doi: 10.3390/ani16111613 (PMC13255817; doi:10.3390/ani16111613)
Supplement: Supplementary file 1 [file animals-16-01613-s001.zip › Table S1.pdf]

Table S1. Summary statistics and counts for categorical and numerical variables: stand's production status, stand's area in hectares, stand's regime, and time since last intervention in days.

Stand.Status	Stand_Size	Regime	T_Intervention
Damaged Production: 27	Min.: 0,00	Afforestation: 50	Min.: 3,0
Forestry Production: 348	1st Qu.: 12,00	Coppice: 222	1st Qu.: 819,5
	Median: 32,00	Reforestation: 103	Median: 1619,0
	Mean: 52,23		Mean: 2011,5
	3rd Qu.: 54,00		3rd Qu.: 3126,5
	Max.: 306,00		Max.: 5628,0
