# Supplementary material for: Eucalyptus Plantation Management Shapes Roe Deer Site-Use Patterns
Source: Animals (Basel). 2026 May 26;16(11):1613. doi: 10.3390/ani16111613 (PMC13255817; doi:10.3390/ani16111613)
Supplement: Supplementary file 1 [file animals-16-01613-s001.zip › Table S2.pdf]

Table S2. Number of camera-trap sites retained in each sampling session and corresponding distribution among the five study areas considered in the occupancy analyses.

Session	Total N sites	Fundão	Pampilhosa	Penamacor	Penha Garcia	Gois
Sess1_Wet_2019	79	22	5	24	18	10
Sess2_Dry_2019	102	25	13	25	22	17
Sess3_Wet_2020	97	24	13	24	23	13
Sess4_Dry_2020	97	21	13	25	23	15
